# GLP-1 Agonists and Cardiovascular Risk Markers in U.S. Adults: National Health and Nutrition Examination Survey (NHANES) 2011-2018

**DOI:** 10.7759/cureus.91722

**Published:** 2025-09-06

**Authors:** B Oneill Telakeng Tekengne, Catherine Ijeoma Eke, Christina Cumaaran, Adaobi A Ozigbo, Chinechem A Okoyeuzu, Inemialu M Okhagbuzo, Rasheedat A Sadiq-Onilenla, Okelue E Okobi

**Affiliations:** 1 Medicine and Surgery, Xuzhou Medical University, Xuzhou, CHN; 2 Family Medicine, University of Port Harcourt Teaching Hospital, Port Harcourt, NGA; 3 Family and Community Medicine, American International School of Medicine, Georgetown, GUY; 4 Internal Medicine, University at Albany, State University of New York, Albany, USA; 5 Internal Medicine, University Hospitals of Derby and Burton NHS Foundation Trust, Derby, GBR; 6 Internal Medicine, Babcock University, Ilishan-Remo, NGA; 7 Health Sciences, Franklin University, Columbus, USA; 8 Family Medicine, Larkin Community Hospital Palm Springs Campus, Hialeah, USA; 9 Family Medicine, IMG Research Academy and Consulting LLC, Homestead, USA

**Keywords:** cardiovascular risk, glp-1 receptor agonists, nhanes, obesity, population-based study, systolic blood pressure

## Abstract

Background and aim: Obesity and overweight remain major contributors to cardiovascular disease (CVD) in the United States. Glucagon-like peptide-1 (GLP-1) receptor agonists, originally developed for glycemic control in type 2 diabetes, have demonstrated cardiometabolic benefits. However, limited population-based evidence exists on their association with cardiovascular risk markers among U.S. adults with overweight or obesity. This study aimed to evaluate the associations between GLP-1 receptor agonist use and cardiovascular risk markers (systolic and diastolic blood pressure, total cholesterol, high-density lipoprotein {HDL} cholesterol, and hemoglobin A1c) in a nationally representative sample of U.S. adults aged ≥18 years with overweight or obesity.

Methods: Data from the National Health and Nutrition Examination Survey (NHANES) 2011-2018 were analyzed using survey-weighted linear regression. Adults aged ≥18 years with overweight or obesity and complete biomarker data were included. Primary exposure was GLP-1 use; outcomes included systolic and diastolic blood pressure, total cholesterol, HDL cholesterol, and hemoglobin A1c.

Results: Among 16,744 unweighted participants, GLP-1 users had significantly lower systolic blood pressure (β = -5.44, p<0.05) compared to non-users. No statistically significant differences were observed in lipid profiles or A1c after adjustment. Age, BMI, insurance status, and diabetes diagnosis were significant covariates.

Conclusion: GLP-1 receptor agonist use was associated with lower systolic blood pressure among adults with overweight or obesity. These findings highlight population-level associations that may inform future research on the cardiometabolic impact of GLP-1 therapies.

## Introduction

The issue of obesity and overweight is pervasive and increasing in the United States, contributing substantially to the burden of chronic diseases, particularly cardiovascular disease (CVD) [1]. The Centers for Disease Control and Prevention (CDC) has indicated that more than 70% of American adults are either obese or overweight, which is directly related to metabolic syndrome and high blood pressure [2,3]. It is also associated with low levels of good cholesterol and high levels of bad cholesterol (dyslipidemia), and the eventual cardiovascular morbidity and mortality [4]. CVD, being the primary cause of death in the United States, is still a top priority in terms of prevention, especially in persons who are more likely to experience cardiovascular adverse events due to their overweight and obese status [5,6].

Conventional management of obesity, including dietary interventions, physical exercise, and behavioral therapy, has not yielded significant long-term success for many patients, primarily due to the biological complexity of weight regulation and behavior [7]. Consequently, pharmacological treatment has become an aspect that is more seriously considered, especially by those who are obese and who have complications [8,9]. Drugs most likely to be helpful in the future include glucagon-like peptide-1 (GLP-1) receptor agonists, a subgroup of drugs initially used to control glycemic levels in patients with type 2 diabetes mellitus (T2DM) [10]. Some GLP-1 agonists, including liraglutide and semaglutide, have recently been approved by the FDA as chronic weight management agents in persons with or without diabetes; this has presented a significant change in clinical utility and population health value [11].

GLP-1 receptor agonists are glucose-dependent insulin secretagogues that act like the incretin hormone GLP-1 to stimulate glucose-stimulated insulin secretion, suppress glucagon release, slow gastric emptying, and increase satiety [12,13]. In addition to their glycemic effects, they have shown similar cardioprotective and weight loss effects in clinical trials and observational studies in recent years [14]. Importantly, in massive cardiovascular outcome trials, e.g., the Liraglutide Effect and Action in Diabetes: Evaluation of Cardiovascular Outcome Results (LEADER), some GLP-1 agonists are found to interfere with adverse cardiovascular outcomes in persons with high baseline cardiovascular risks [15].

Although such strong evidence exists, it has been found that most of the existing literature concentrates on patients with diabetes or high-risk individuals in the clinical trial scenario, usually missing population-based research [16]. However, few studies have discussed the use of GLP-1 agonists in association with overweight or obese people without diabetes, as well as examining the pattern of the interventions, their association with quantifiable cardiovascular risk factors in the general adult population in the United States [17,18].

This knowledge gap, which is addressed by the study, highlights the importance of placebo-controlled outcomes in analyses at the population level rather than restricted to clinical trial definitions to determine cardiovascular implications of using GLP-1 agonists more broadly [19]. The National Health and Nutrition Examination Survey (NHANES) provides an abundant source of health determinants that are nationally representative, not restricted to self-reported use of prescription medication, details of anthropometric measures, and biochemical markers pertinent to cardiovascular health [20]. The research aimed to examine the potentially existing association between the introduction of GLP-1 receptor agonists and cardiovascular disease (CVD) major risk indicators among adult citizens of the United States with overweight or obesity, based on the 2011-2018 dataset of the NHANES study.

## Materials and methods

Study design and data source

This study employed a cross-sectional design, utilizing data from the National Health and Nutrition Examination Survey (NHANES) for the years 2011-2018. NHANES was conducted by the National Center for Health Statistics (NCHS) and employs a complex, multistage probability sampling design to obtain a nationally representative sample of the non-institutionalized U.S. population. Data were collected through household interviews, physical examinations, and laboratory testing. NHANES is publicly available and widely used in epidemiological research, offering robust datasets ideal for evaluating population health patterns and medication use [21].

Study population

The study population included adults aged 18 years and older who had a body mass index (BMI) classified as overweight or obese (BMI ≥25 kg/m²). Individuals were eligible if they had complete data on prescription medication use and cardiovascular risk markers.

Exposure variable

The primary exposure of interest was the use of glucagon-like peptide-1 (GLP-1) receptor agonists. These medications were identified from the prescription medication component of NHANES, in which participants reported all prescription drugs taken in the past 30 days. Each medication entry included both a drug name (RXDDRUG) and a standardized drug identifier (RXDRGID) derived from the Lexicon Plus database. Participants were classified as GLP-1 users if they reported using any of the following medications: exenatide (d05529), liraglutide (d07466), dulaglutide (d08290), or semaglutide (d08688). These drugs represent the most commonly prescribed GLP-1 receptor agonists during the study period. All other participants with available prescription data who did not report using any of these medications were categorized as non-users.

Outcome variables

Cardiovascular disease (CVD) risk markers included systolic and diastolic blood pressure, total cholesterol, high-density lipoprotein (HDL) cholesterol, fasting glucose, and hemoglobin A1c. These measures are routinely collected in the NHANES laboratory and examination components and are widely recognized indicators of cardiometabolic risk. Where multiple blood pressure readings were available, the mean of the first three non-missing values was used. Fasting glucose was analyzed only among participants from the fasting subsample. Hemoglobin A1c, available for the full sample, was included as a stable marker of long-term glycemic status.

Covariates

To account for potential confounding, the following covariates were included in the models: age (categorized), sex, race/ethnicity, educational attainment, health insurance status, smoking status, physical activity level, diabetes diagnosis, hypertension status, and BMI. These variables were selected based on prior literature indicating associations with both GLP-1 use and cardiovascular outcomes.

Statistical analysis

Descriptive statistics were calculated to compare demographic and clinical characteristics between GLP-1 users and non-users. Differences between groups were assessed using survey-weighted chi-square tests for categorical variables and t-tests for continuous variables. Survey-weighted linear regression models were used to examine the association between GLP-1 use and each continuous cardiovascular risk marker.

All analyses incorporated NHANES-provided sample weights, strata, and primary sampling units (PSUs) to account for the complex survey design and to ensure nationally representative estimates. Statistical significance was defined as a two-tailed p<0.05. Analyses were conducted using Stata version 18.0 (College Station, TX: StataCorp LLC.).

Missing data

In this study, missing data were retained rather than excluded to preserve statistical power, particularly because the number of GLP-1 receptor agonist users in the sample (n=78) was minimal. The original pooled sample from NHANES 2011-2018 included 16,744 adults, and the primary exposure variable, GLP-1 agonist use, had no missing data (0%). Most covariates also exhibited low levels of missingness, including education level (1.92%), smoking status (0.51%), hypertension status (0.10%), and insurance coverage (0.24%). However, several continuous outcome variables had moderately higher proportions of missing data, including systolic and diastolic blood pressure (9.91%), BMI (7.91%), HbA1c (10.03%), total cholesterol and HDL cholesterol (each 11.16%), and income-to-poverty ratio (10.95%). Despite these levels of missingness, multiple imputation techniques were not used due to incompatibility with the complex NHANES survey weighting design in Stata. Therefore, a survey-weighted complete-case analysis approach was applied, using available data per model while maintaining consistency and representativeness of the national population.

Ethical considerations

NHANES data are de-identified and publicly available. As this study used secondary, publicly available data without direct involvement of human subjects, no additional institutional review board (IRB) approval was required.

## Results

Table 1 summarizes the baseline characteristics of adults reporting GLP-1 receptor agonist use (n=802,037) compared with non-users (n=166,848,239), based on NHANES 2011-2018 survey-weighted data.

**Table 1:** Baseline characteristics of U.S. adults by GLP-1 receptor agonist use, NHANES 2011-2018 (unweighted sample: n=16,744; weighted population estimates: GLP-1 users=802,037; non-users=166,848,239). P-value <0.05 is statistically significant. This table presents weighted descriptive statistics comparing cardiovascular risk markers and socioeconomic and demographic characteristics between adults who reported GLP-1 receptor agonist use and those who did not, using NHANES data from 2011 to 2018. The unweighted analytic sample included 16,744 adults, of whom a small subset reported GLP-1 use. Weighted counts represent population-level estimates after accounting for the NHANES complex survey design. Continuous variables are presented as weighted means with standard deviations, while categorical variables are presented as weighted counts and percentages. Statistical differences were evaluated using design-based F- or t-tests. Education level is reported only for participants aged ≥20 years, consistent with NHANES data collection. NHANES: National Health and Nutrition Examination Survey; NA: no data for the variable; GED: General Educational Development

Variables	GLP-1 users (n=802,037)	GLP-1 non-users (n=166,848,239)	Design-based F/t-test	p-Value
Systolic BP (mmHg), (mean±SD)	123.58±13.93	124.03±16.54	t=0.22	0.827
Diastolic BP (mmHg), (mean±SD)	68.66±9.94	71.83±12.03	t=2.22	0.030
Direct HDL-cholesterol (mg/dL), (mean±SD)	44.58±11.20	50.72±15.00	t=3.95	p<0.001
Total cholesterol (mg/dL), (mean±SD)	170.24±37.12	194.11±42.23	t=3.38	0.001
Age (years), (mean±SD)	52.72±8.21	46.24±16.83	t = -5.57	p<0.001
Glycohemoglobin (%), (mean±SD)	7.69±1.54	5.73±1.01	t = -8.99	p<0.001
Family poverty income ratio, (mean±SD)	3.38±1.62	2.98±1.65	t = -1.46	0.151
Body mass index (kg/m^2^), (mean±SD)	36.56±8.43	32.11±6.16	t = -3.63	0.001
Gender (%)				
Male	406,600 (0.48%)	83,731,192 (99.52%)	F=0.004	0.951
Female	395,436 (0.47%)	83,117,046 (99.53%)		
Race/ethnicity (%)				
Mexican American	41,305 (0.24%)	16,961,799 (99.76%)	F=2.18	0.09
Other Hispanic	26,145 (0.23%)	11,396,295 (99.77%)		
Non-Hispanic White	580,846 (0.54%)	106,343,232 (99.46%)		
Non-Hispanic Black	125,957 (0.62%)	20,278,504 (99.38%)		
Other race	27,782 (0.23%)	11,868,407 (99.77%)		
Education level (adults 20+ years) (%)				
Less than 9th grade	26,574 (0.29%)	9,009,364 (99.71%)	F=0.95	0.419
9-11th grade	47,062 (0.30%)	15,534,282 (99.70%)		
High school grad/GED	182,270 (0.47%)	38,904,668 (99.53%)		
Some college or AA degree	233,758 (0.42%)	54,821,503 (99.58%)		
College graduate or above	312,371 (0.66%)	46,732,770 (99.34%)		
Health insurance coverage (%)				
Uninsured	7,649 (0.03%)	27,012,788 (99.97%)	F=17.44	p<0.001
Insured	794,387 (0.57%)	139,548,789 (0.99%)		
Moderate-to-vigorous physical activity (MVPA) in days (%)				
1-2 days	115,826 (0.38%)	30,571,897 (99.62%)	F=0.42	0.614
3-5 days	686,210 (0.50%)	136,203,906 (99.50%)		
≥5 days	NA	72,435 (100%)		
Doctor told you have diabetes (%)				
Yes	783,705 (3.62%)	20,888,374 (96.38%)	F=271.66	p<0.001
No	18,331 (0.01%)	141,366,492 (99.99%)		
Ever told by doctor that you have hypertension (%)				
Yes	560,045 (0.87%)	63,765,262 (99.13%)	F=14.56	p<0.001
No	241,991 (0.23%)	102,941,431 (99.77%)		
Smoked at least 100 cigarettes in life (%)				
Yes	447,988 (0.61%)	72,977,843 (99.39%)	F=2.37	0.129
No	354,048 (0.38%)	93,393,805 (99.62%)		

From Table 1 above, it’s evident that the mean systolic blood pressure was similar between GLP-1 users (123.58±13.93 mmHg) and non-users (124.03±16.54 mmHg), with no significant difference (p=0.827). However, mean diastolic blood pressure was significantly lower among GLP-1 users (68.66±9.94 mmHg) compared to non-users (71.83±12.03 mmHg; p=0.030).

GLP-1 users had markedly lower mean total cholesterol levels (170.24±37.12 mg/dL) than non-users (194.11±42.23 mg/dL; p=0.001), and also had significantly lower mean HDL cholesterol (44.58±11.20 mg/dL) compared to non-users (50.72±15.00 mg/dL; p<0.001). Additionally, mean HbA1c levels were substantially higher in GLP-1 users (7.69±1.54%) than in non-users (5.73±1.01%; p<0.001). This difference is expected, as GLP-1 receptor agonists are most commonly prescribed for individuals with diabetes, whereas the non-user group includes many adults without diabetes. GLP-1 users were also older (mean age: 52.72±8.21 years) than non-users (mean age: 46.24±16.83 years; p<0.001) and had a higher mean BMI (36.56±8.43 kg/m² vs. 32.11±6.16 kg/m²; p=0.001). The family income-to-poverty ratio did not differ significantly between groups (p=0.151).

In terms of categorical characteristics, gender distribution was balanced between groups, with 406,600 (0.48%) males and 395,436 (0.47%) females among GLP-1 users, and 83,731,192 (99.52%) males and 83,117,046 (99.53%) females among non-users (p=0.951). Racial and ethnic distributions did not significantly differ between groups (p=0.09). The majority of GLP-1 users were non-Hispanic White (580,846; 0.54%) or non-Hispanic Black (125,957; 0.62%), with smaller proportions of Mexican American (41,305; 0.24%), other Hispanic (26,145; 0.23%), and other race (27,782; 0.23%).

Educational attainment also did not differ significantly by GLP-1 use (p=0.419). Among users, 26,574 (0.29%) had less than a 9th-grade education, 47,062 (0.30%) completed 9th-11th grade, 182,270 (0.47%) were high school graduates or had a GED, 233,758 (0.42%) had some college or an associate degree, and 312,371 (0.66%) had a college degree or higher. A greater proportion of GLP-1 users were insured (794,387; 0.57%) compared to non-users (139,548,789; 0.99%), and insurance coverage was significantly associated with GLP-1 use (p<0.001).

There was no significant difference in physical activity levels (p=0.614). Among GLP-1 users, 115,826 (0.38%) reported one to two days of moderate-to-vigorous physical activity per week, and 686,210 (0.50%) reported three to five days. Diabetes status strongly differed by GLP-1 use (p<0.001), with 783,705 (3.62%) GLP-1 users having been told they have diabetes, compared to 20,888,374 (96.38%) of non-users. Hypertension diagnosis was also more common in the GLP-1 group (560,045; 0.87%) compared to non-users (63,765,262; 99.13%; p<0.001). Smoking history did not significantly differ (p=0.129), with 447,988 (0.61%) GLP-1 users and 72,977,843 (99.39%) non-users reporting having smoked at least 100 cigarettes in their lifetime. Table 2 presents the multivariable regression results examining the independent associations between GLP-1 receptor agonist use and continuous cardiovascular risk markers.

**Table 2:** Association between GLP-1 receptor agonist use and cardiovascular risk markers in U.S. adults, NHANES 2011-2018. *P-value <0.05 is statistically significant. **P-value <0.001 is statistically significant. ***P-value <0.01 is statistically significant. Survey-weighted linear regression models were used to examine the association between GLP-1 receptor agonist use and five cardiovascular disease (CVD) risk markers as follows: systolic blood pressure, diastolic blood pressure, total cholesterol, HDL cholesterol, and hemoglobin A1c. Models adjusted for age, sex, BMI, income-to-poverty ratio, insurance status, diabetes status, hypertension diagnosis, physical activity level, smoking, education, and race/ethnicity. Education level is reported only for participants aged ≥20 years, consistent with NHANES data collection. Standard errors are presented in parentheses. NHANES: National Health and Nutrition Examination Survey; GED: General Educational Development

Predictors	Systolic BP (mmHg)	Diastolic BP (mmHg)	Total cholesterol (mmHg)	HDL cholesterol (mmHg)	Hemoglobin A1c
GLP-1 agonist use (SE)	-5.444^* ^(2.288)	-2.141 (1.482)	-8.102 (7.093)	-1.435 (2.075)	0.390 (0.248)
Age (years) (SE)	0.327^** ^(0.012)	-0.060^** ^(0.010)	0.238^** ^(0.034)	0.123^** ^(0.013)	0.010^** ^(0.001)
Body mass index (kg/m^2^) (SE)	0.330^** ^(0.030)	0.186^** ^(0.022)	-0.310^** ^(0.087)	-0.444^** ^(0.030)	0.016^** ^(0.002)
Ratio of family income to poverty (SE)	-0.111 (0.127)	0.509^** ^(0.132)	1.486^** ^(0.374)	0.862^** ^(0.123)	-0.009 (0.007)
Gender (reference=female) (SE)	-3.531^** ^(0.329)	-3.202^** ^(0.286)	8.880^** ^(1.033)	10.906^** ^(0.347)	-0.041^* ^(0.017)
Health insurance (reference=yes) (SE)	-1.315^*** ^(0.484)	-0.948 (0.490)	-5.119^** ^(1.385)	0.882^* ^(0.378)	-0.070^*** ^(0.023)
Doctor told you have diabetes (reference=no) (SE)	1.572^* ^(0.652)	3.133^** ^(0.420)	17.698^** ^(1.437)	5.135^** ^(0.432)	-1.701^** ^(0.049)
Doctor told you have hypertension (reference=yes) (SE)	6.838^** ^(0.425)	2.554^** ^(0.292)	-2.394^* ^(1.056)	-0.989^* ^(0.390)	0.005 (0.018)
Moderate-to-vigorous physical activity (MVPA) in days					
3-4 days (SE)	-1.087^* ^(0.489)	-1.039^** ^(0.355)	-3.657^* ^(1.539)	-0.704 (0.376)	-0.015 (0.022)
≥5 days (SE)	-8.240 (4.298)	-0.113 (4.102)	-3.218 (12.707)	-6.614^* ^(3.075)	0.703 (0.516)
Smoked ≥100 cigarettes in life (reference=no) (SE)	0.190 (0.441)	0.632^* ^(0.297)	-3.488^** ^(1.071)	0.072 (0.304)	-0.045^* ^(0.018)
Education level (adults 20+ years)					
9-11th grade (SE)	-0.309 (0.633)	0.758 (0.548)	0.987 (1.830)	-0.711 (0.610)	-0.094^* ^(0.046)
High school grade/GED (SE)	-0.623 (0.788)	0.956 (0.661)	-0.751 (1.944)	0.049 (0.665)	-0.078^* ^(0.038)
Some college or AA degree (SE)	-1.460^* ^(0.707)	1.179 (0.648)	-0.081 (1.685)	0.266 (0.659)	-0.116^** ^(0.039)
College graduate or above (SE)	-2.313^** ^(0.746)	0.976 (0.721)	-1.978 (2.125)	1.775^* ^(0.686)	-0.093^* ^(0.042)
Race/ethnicity					
Other Hispanic (SE)	0.379 (0.476)	-0.037 (0.678)	-1.378 (1.814)	-0.719 (0.438)	-0.014 (0.036)
Non-Hispanic White (SE)	-0.390 (0.431)	0.543 (0.477)	0.098 (1.541)	0.465 (0.392)	-0.203^** ^(0.030)
Non-Hispanic Black (SE)	4.005^** ^(0.489)	1.291^* ^(0.548)	-4.192^* ^(1.588)	4.984^** ^(0.525)	0.056 (0.033)
Other race (SE)	1.471^* ^(0.669)	2.162^** ^(0.433)	0.469 (1.567)	-1.874^** ^(0.563)	0.024 (0.044)
Constant (SE)	98.583^** ^(1.613)	64.876^** ^(1.197)	179.768^** ^(4.018)	45.579^** ^(1.387)	6.586^** ^(0.089)
R^2^	0.210	0.055	0.045	0.212	0.429

After adjusting for demographic, clinical, and behavioral covariates, GLP-1 receptor agonist use was significantly associated with lower systolic blood pressure, with an average reduction of 5.44 mmHg (SE=2.29; p<0.05). However, GLP-1 use was not significantly associated with diastolic blood pressure, which showed a smaller and non-significant decrease of 2.14 mmHg (SE=1.48; p>0.05).

In terms of lipid profiles, GLP-1 users had lower mean total cholesterol by 8.10 mg/dL (SE=7.09), though this was not statistically significant. Similarly, HDL cholesterol was lower among GLP-1 users by 1.44 mg/dL (SE=2.08), but this finding was also not statistically significant. Interestingly, GLP-1 use was associated with a modest increase in hemoglobin A1c, with an adjusted mean difference of 0.39 percentage points (SE=0.25), although this increase was not statistically significant (p>0.05). The elevated HbA1c levels likely reflect the underlying diabetic status that prompts GLP-1 therapy.

Several covariates were also significantly associated with cardiovascular outcomes. Age was positively associated with systolic BP, total cholesterol, HDL cholesterol, and HbA1c, but inversely associated with diastolic BP. Higher BMI was associated with increased systolic and diastolic BP and HbA1c, but lower total and HDL cholesterol. Males had significantly lower blood pressure but higher cholesterol and HDL levels than females. Uninsured individuals had significantly lower systolic BP and total cholesterol, but higher HDL and lower HbA1c values than those with insurance.

A diagnosis of diabetes was associated with higher levels of all risk markers except HDL cholesterol. Hypertension diagnosis was associated with substantially higher systolic and diastolic BP, while also correlating with lower total and HDL cholesterol levels. Among lifestyle factors, higher levels of moderate-to-vigorous physical activity (MVPA) showed a protective association, particularly with systolic BP and HDL cholesterol. Smoking history was associated with lower total cholesterol and higher diastolic BP. Education level demonstrated mixed associations, with higher education generally linked to more favorable lipid and glycemic profiles. Race/ethnicity was also significantly associated with multiple outcomes. For instance, non-Hispanic Black participants had higher systolic and diastolic BP, but lower total cholesterol, and higher HDL compared to other groups.

The adjusted R² values indicated that the models explained 21.0% of the variance in systolic BP, 5.5% in diastolic BP, 4.5% in total cholesterol, 21.2% in HDL cholesterol, and 42.9% in HbA1c, suggesting a stronger explanatory power, particularly for glycemic control.

## Discussion

This study evaluated the association between GLP-1 receptor agonist use and key cardiovascular disease (CVD) risk markers, namely blood pressure, lipid profile, and glycemic control, among U.S. adults with overweight or obesity, using nationally representative NHANES data from 2011 to 2018. The findings contribute novel population-level evidence to an area largely dominated by clinical trials and diabetes-centric studies, addressing a key research gap identified in prior literature [16-18].

In adjusted models, GLP-1 receptor agonist use was significantly associated with a reduction in systolic blood pressure by approximately 5.4 mmHg, aligning with clinical evidence that these agents exert mild antihypertensive effects [14,15]. There are two main mechanisms used by GLP-1 receptor agonists like semaglutide to regulate blood pressure as follows: the first mechanism is through the improvement of vascular endothelial function, which results in a reduction of oxidative stress. The second mechanism is the dilation of blood vessels. The FLOW trial demonstrated that a weekly 1.0 mg dose of semaglutide can reduce major kidney events by up to 24% compared to placebo. GLP-1 receptor agonists can also lower the blood pressure by inhibiting sodium-hydrogen exchanger 3 (NHE3) in the proximal convoluted tubule, therefore promoting sodium excretion and concomitantly lowering blood pressure [14,15].

While the reduction in diastolic pressure was not statistically significant, the trend was directionally consistent. These results support earlier cardiovascular outcome trials, such as the Liraglutide Effect and Action in Diabetes: Evaluation of Cardiovascular Outcome Results (LEADER) trial, where liraglutide was associated with reduced cardiovascular events [15]. However, unlike tightly controlled trial settings, our population-based data reflect real-world pharmacological exposure, including diverse clinical backgrounds and usage patterns.

Contrary to initial expectations, GLP-1 use was not associated with significant improvements in lipid markers. Both HDL cholesterol and total cholesterol showed slightly lower mean levels among users, but the differences were not statistically significant after covariate adjustment. This finding diverges somewhat from prior meta-analyses that suggested modest HDL improvements with GLP-1 agonists [17,18]. The discrepancy may stem from shorter exposure duration, lower adherence, or overlapping use of statins and other lipid-lowering medications in the general population.

Unexpectedly, GLP-1 users had marginally higher levels of hemoglobin A1c than non-users, though this difference did not reach statistical significance. This paradoxical finding likely reflects confounding by indication; GLP-1 agonists are primarily prescribed for individuals with poorly controlled type 2 diabetes, which itself drives elevated HbA1c values despite therapy. Thus, while GLP-1 use may reduce A1c over time, users tend to begin treatment at higher glycemic baselines.

Additional covariates showed consistent relationships with cardiovascular risk markers. Higher age and BMI were positively associated with systolic BP, HbA1c, and cholesterol abnormalities, echoing well-documented metabolic trends in obesity-related cardiovascular disease [1-3,5]. Notably, individuals with health insurance and higher educational attainment tended to exhibit more favorable profiles, suggesting that access to care and health literacy continue to mediate chronic disease outcomes. These patterns reinforce structural health determinants emphasized in national prevention strategies [6]. The impact of BMI on the pharmacologic response to GLP-1 receptor agonists is likely driven by metabolic and physiological differences linked to excess adiposity. Individuals with higher BMI often exhibit greater weight loss, partly due to the dose-dependent effects of these medications [9]. Moreover, obesity-related factors, such as altered insulin sensitivity, chronic inflammation, and disrupted energy regulation, may influence drug responsiveness. These considerations underscore the need to pair GLP-1 therapy with lifestyle strategies, particularly regular physical activity, to maximize weight reduction and enhance cardiometabolic outcomes in patients with elevated BMI [12].

From a behavioral standpoint, higher frequency of moderate-to-vigorous physical activity was associated with lower systolic pressure and improved lipid profiles, consistent with established guidelines on physical activity and cardiovascular health [7]. Smoking, by contrast, was associated with lower total cholesterol but higher diastolic pressure, an expected yet complex relationship previously observed in cardiovascular epidemiology.

Sustained cardiovascular benefits, however, can be undermined by weight regain, a risk that can be mitigated through moderate to vigorous physical activity. Such activity not only helps preserve lean muscle mass but also supports metabolic rate by improving lipid and glucose regulation. This, in turn, enhances the impact of dietary interventions, improves functional capacity, and promotes long-term adherence to healthy behaviors [8]. Evidence increasingly suggests that the effectiveness of pharmacologic treatments is amplified when integrated with consistent physical activity over the long term.

Limitations and strengths of the study

This study has several limitations worth noting. First, the relatively low number of participants using GLP-1 receptor agonists (n=78 out of 16,744) may have limited the statistical power to detect small but clinically meaningful associations. This small exposure group could have contributed to wider confidence intervals and reduced generalizability for GLP-1 users in subgroup analyses. Second, because younger adults in the sample are more likely to have normal blood pressure, this may attenuate the observed associations; however, the NHANES survey design weights the data to represent the full U.S. adult population with overweight or obesity, including both younger and older adults.

Although survey-weighted techniques were employed to account for NHANES’s complex sampling design, certain levels of missing data in outcome variables, such as HbA1c (10.03%), cholesterol measures (11.16%), and blood pressure (9.91%), could introduce bias if the missingness was not completely at random. Multiple imputation was not feasible due to incompatibility with the survey weighting structure in Stata.

Nonetheless, this study has several notable strengths. The use of nationally representative NHANES data from 2011 to 2018 ensures broad generalizability to the United States adult population. Unlike many previous studies, this analysis included all four major GLP-1 receptor agonists approved during the study period as follows: exenatide, liraglutide, dulaglutide, and semaglutide, capturing real-world medication use. Additionally, the availability of objective biochemical and clinical measurements allowed for a robust assessment of cardiovascular risk markers.

Despite their wide range of benefits, GLP-1 receptor agonists are still disproportionately prescribed even in cases of serious events like type 2 diabetes (T2D). Asian, Hispanic, and Black individuals have significantly lower access. Higher income has been associated with better access, the reverse being true for low-income earners. Gender, insurance type, and geographic distribution affect access to GLP-1 receptor agonists [22,23].

Future studies should consider larger pooled datasets or targeted sampling to increase the number of GLP-1 users, thereby providing more precise estimates. Prospective designs or longitudinal population-based cohorts may help clarify causal relationships and monitor changes in cardiovascular outcomes over time.

## Conclusions

In this nationally representative analysis of U.S. adults with overweight or obesity, GLP-1 receptor agonist use was associated with significantly lower systolic blood pressure but showed no significant improvement in lipid levels or glycemic markers after adjustment. These findings support the potential cardiovascular benefit of GLP-1 agonists beyond glycemic control, particularly for blood pressure reduction. Continued monitoring and population-level research are essential to understand their impact on non-diabetic and diverse real-world populations fully.
